# A novel and robust pyroptosis-related prognostic signature predicts prognosis and response to immunotherapy in esophageal squamous cell carcinoma

**DOI:** 10.18632/aging.204946

**Published:** 2023-08-09

**Authors:** Dengfeng Zhang, Fangchao Zhao, Jing Li, Xuebo Qin, Shujun Li, Ren Niu

**Affiliations:** 1 Department of Thoracic Surgery, The Second Hospital of Hebei Medical University, Shijiazhuang, China; 2 Department of Thoracic Surgery, Hebei Chest Hospital, Shijiazhuang, China; 3 Department of Oncology, The Second Hospital of Hebei Medical University, Shijiazhuang, China

**Keywords:** pyroptosis, esophageal squamous cell carcinoma, prognosis, tumor microenvironment, immunotherapy

## Abstract

Esophageal squamous cell carcinoma (ESCC) is a highly malignant gastrointestinal tumor, has a poor prognosis and high mortality rate. Pyroptosis could regulate tumor cell proliferation, invasion, and metastasis, thereby affecting the prognosis of cancer patients. However, the role of pyroptosis-related genes (PRGs) in ESCC remains unclear. This study selected 33 PRGs, and finally identified 29 PRGs that were differentially expressed between ESCC and normal esophageal tissues. The genetic mutation variation landscape of PRG in ESCC was also summarised. Based on consensus clustering for the 33 PRGs, all ESCC patients could be divided into two subtypes. Functional enrichment analysis revealed that these 33 PRGs were mainly involved in cytokine production, interleukin-1 production, and the NOD-like receptor signalling pathway. We created a prognostic PRG signature based on least absolute shrinkage and selection operator regression and Cox regression analysis with good survival prediction ability in both GEO and TCGA cohorts. Combined with the clinical characteristics, signature–based risk score was found to be an independent factor for predicting the OS of ESCC patients. A nomogram with enhanced precision for forecasting ESCC was established based on various independent prognostic elements. Significant correlation was observed between prognostic PRGs and immune-cell infiltration, tumor mutation burden, microsatellite instability, immune checkpoint, and drug sensitivity. Finally, we validated the expression of four PRGs in ESCC cell lines and tissues samples. In conclusion, the PRGs exerted significant effects on tumor immunity and prognosis of ESCC.

## INTRODUCTION

As a common malignant digestive system cancer, esophageal carcinoma (ESCA) was number 8 in morbidity and number 6 in mortality across the world [1]. On basis of the National Central Cancer Registry of China (NCCR) statistics, Chinese ESCA patients comprise up to 70% of all ESCA cases across the world [2]. Unlike the European and American populations where adenocarcinoma predominates, over 90% of Chinese patients with ESCA are esophageal squamous cell carcinoma (ESCC) [3]. Standardized surgical intervention stands as the primary therapeutic approach for addressing esophageal cancer. Nevertheless, solitary reliance on surgical procedures frequently fails to achieve a comprehensive remission for individuals afflicted by locally advanced esophageal malignancies [4]. Over the years, extensive research has been conducted on various modalities including radiotherapy, chemotherapy, targeted therapy, and biological therapy for the management of esophageal cancer. Despite these efforts, the somber reality persists: the 5-year survival rate for individuals afflicted by esophageal cancer remains distressingly low, lingering below the threshold of 20% [5, 6]. Consequently, it is imperative to expeditiously identify novel, highly sensitive biomarkers capable of prognosticating the survival outcomes of patients diagnosed with esophageal squamous cell carcinoma (ESCC).

Pyroptosis, a form of programmed cell death mediated by inflammasomes, is characterized by a relentless cellular expansion that culminates in the rupture of the cell membrane, subsequently releasing cellular contents and inflammatory mediators. This dramatic event incites a robust inflammatory cascade, thereby eliciting a potent inflammatory response [7, 8]. The occurrence of pyroptosis depends on the cysteinyl aspartate-specific proteinase (caspase) and the gasdermin (GSDM) family proteins. Following activation by caspases, the hinge region linking the N-terminal and C-terminal domains of gasdermin D (GSDMD) or gasdermin E (GSDME) undergoes cleavage, liberating a segment endowed with potent cytotoxic properties. This pivotal cleavage event ultimately leads to the induction of pyroptosis [9]. The intricate relationship between pyroptosis and cancer is multifaceted. On one hand, the pivotal inflammasomes involved in pyroptosis have demonstrated the ability to stimulate tumor cell death, impede tumor cell proliferation, and suppress metastatic potential [10, 11]. On the other hand, the accumulation of inflammasomes within the tumor microenvironment creates a conducive milieu that facilitates tumor cell proliferation, invasion, and metastasis. Consequently, this promotes the rapid progression of tumor growth [12, 13]. A burgeoning body of evidence has illuminated the significant impact of pyroptosis on cancer prognosis, specifically through its profound influence on tumor cell proliferation, invasion, and metastasis. These studies underscore the pivotal role of pyroptosis in shaping the clinical outcomes of various types of cancer [14]. For prognostic purposes, a novel gene signature associated with pyroptosis, termed the Pyroptosis-Related Gene (PRG) signature, has recently emerged in ovarian cancer, gastric cancer, and lung adenocarcinoma. This signature holds promise as a valuable tool for predicting the clinical outcomes of patients afflicted with these particular malignancies [15–17]. However, there is no research that clarifies the effect of PRGs on ESCC, and our research was conducted to illustrate this effect.

In light of this research gap, we have successfully developed a pioneering prognostic signature for esophageal squamous cell carcinoma (ESCC) utilizing four carefully selected Pyroptosis-Related Genes (PRGs). Through rigorous validation, we have established the robustness and reliability of this signature, potentially offering a novel avenue for accurately predicting clinical outcomes and facilitating the tailored selection of therapeutic strategies. This groundbreaking approach holds promising implications for advancing personalized treatment strategies in ESCC patients.

## MATERIALS AND METHODS

### Dataset and preprocessing

The transcriptome data, obtained from The Cancer Genome Atlas (TCGA) database, encompassed RNA-seq profiles of esophageal squamous cell carcinoma (ESCC) patients, which were quantified using the Fragment Per Kilobase method (FPKM). This dataset consisted of 80 ESCC tissue samples and 11 corresponding normal tissue samples. Additionally, to enhance the comprehensiveness of our study, we retrieved additional RNA-seq data from the Gene Expression Omnibus (GEO) database, specifically the dataset GSE53624, which included 119 normal tissue samples and 119 tumor tissue samples. In parallel, somatic datasets and copy number variation (CNV) data were acquired from the TCGA database, further enriching our analysis with comprehensive genetic information.

### Mutation and differential expression analysis of PRGs

To explore the characteristics of the 33 Pyroptosis-Related Genes (PRGs) in esophageal squamous cell carcinoma (ESCC) patients, several analytical methods were employed. Firstly, the “maftools” package was utilized to generate mutation frequency plots and an oncoplot waterfall plot, illustrating the distribution and frequency of mutations within the PRGs across the ESCC patient cohort. Furthermore, the “RCircos” package in R was employed to visualize the location of copy number variation (CNV) alterations of the 33 PRGs on the 23 chromosomes. To investigate the differential expression of PRGs between ESCC and normal tissues, the “limma” and “reshape2” packages were employed. These tools enabled the identification of statistically significant differences in PRG expression levels. In order to gain insights into potential protein-protein interactions among the 33 PRGs, a protein-protein interaction (PPI) network was constructed using the Search Tool for the Retrieval of Interacting Genes (STRING) database. This network analysis helped unravel the interconnections and functional associations among the PRGs, providing valuable information about their collective behavior in ESCC.

### Functional enrichment analysis

The process of extracting Gene Ontology (GO) analysis and conducting enrichment analysis of the Kyoto Encyclopedia of Genes and Genomes (KEGG) from the output generated by the “clusterProfiler” package was performed. In addition, single-sample gene set enrichment analysis (ssGSEA) method was adopted to differentiate patients sorted by risk score [18].

### Construction of prognostic risk model and nomogram

The prognostic significance of PRGs was evaluated using Cox regression analysis. To ensure no omission, 0.1 was set as the cut-off *P*-value, and further analysis selected four PRGs with a great prognostic value. In order to identify genes with prognostic significance, a least absolute shrinkage and selection operator (LASSO) regression approach was employed for screening purposes. Subsequently, prognostic models were constructed through multivariate regression analysis using the selected genes [19]. The risk score calculating formula is:


$$\sum_{i=1}^{n}{\text{Coef}}_{i}\;\times \;x_{i}$$

The variables Coef_*i*_ and *x_i_* denote the coefficient and expression levels of the respective gene, respectively. Based on the median risk score, the ESCC patients from the Gene Expression Omnibus (GEO) and The Cancer Genome Atlas (TCGA) databases were stratified into low- and high-risk subgroups. Subsequently, the overall survival (OS) time was compared between these two subgroups using Kaplan-Meier analysis. A receiver operating characteristic (ROC) curve analysis was conducted with the “survival”, “survminer”, and “timeROC” R packages. Univariate and multivariate Cox regression models were used to analyse these variables in combination with the risk scores. Utilizing the coefficients obtained from the aforementioned formula, we employed the “rms” package to construct a nomogram. Additionally, to validate the predictive performance of the nomogram, we conducted receiver operating characteristic (ROC) analysis and calibration curve analysis.

### Comprehensive analysis about immune cell infiltration

TIMER, XCELL, QUANTISEQ, MCPCOUNTER, EPIC, CIBERSORTABS, and CIBERSORT R script were applied to quantify the relative proportions of infiltrating immune cells [20]. In addition, the “gsva” package was employed to evaluate the activity of immune-related pathways using ssGSEA. The expression levels of immune checkpoint-associated genes may be associated with treatment responses to immune checkpoint inhibitors. The differences in gene expression levels between the high-risk and low-risk groups were then tested to determine the relationship between the risk score and response to immune checkpoint inhibitors. Similarly, we also investigated the disparities in microsatellite instability (MSI) and tumor mutation burden (TMB) among the distinct risk groups.

### Tissue samples and quantitative real-time polymerase chain reaction (qRT-PCR)

In this study, a cohort of ESCC patients who underwent tumor resection provided a total of 10 tumor tissue samples along with their corresponding adjacent normal esophageal tissue samples. These samples were collected from the Thoracic Surgery Department of the Second Hospital of Hebei Medical University, following the approval of the study by the Medical Ethics Committee of the hospital. Eca109, TE-1, HET-1A, and KYSE150 cells were obtained from the Chinese Academy of Sciences (Shanghai, China). All cell lines were cultured in RPMI 1640 medium with 10% fetal bovine serum (FBS) and incubated at 37°C with 5% CO_2_. Medium and FBS were purchased from Corning. Total RNA was extracted using TRIzol reagent (Invitrogen, USA), and an qRT-PCR kit (Bestar; DBI Bioscience) was used to synthesise cDNA. qRT-PCR was performed using the Stratagene RT-PCR system (Applied Biosystems, USA). Relative gene expression was quantified via the 2^−ΔΔCT^ method. GAPDH acted as the internal reference for normalization.

To facilitate the interpretation of results across different platforms, the scores obtained from tissue samples were further standardized and simplified, resulting in the generation of a riskscore. The riskscore was derived by mapping the score values, which involved subtracting the minimum value and dividing by the maximum value. This mapping process allowed for a consistent and comparable interpretation of the riskscore. The calculation of the riskscore using qRT-PCR was performed according to the following formula: Riskscore (qRT-PCR) = (Score − Min)/Max.

### Statistical analysis

All statistical analyses were conducted using R software (version 4.0.1). Detailed information regarding the statistical methods employed for the analysis of transcriptome data can be found in the bioinformatics method section. A significance level of *P* < 0.05 was utilized to determine statistical significance.

## RESULTS

### Mutation landscape of PRGs in ESCC

**Figure 1:**
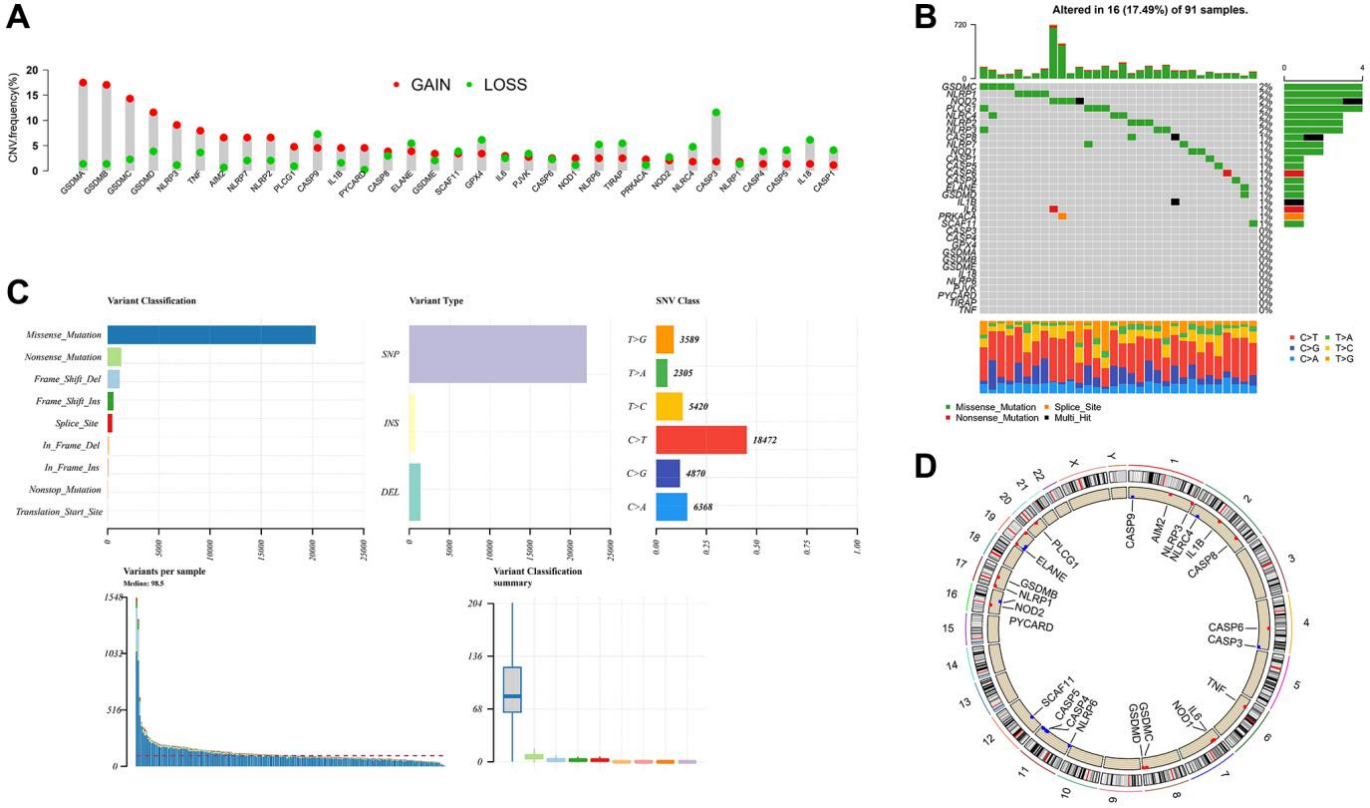
**Mutation landscape of PRG in ESCC.** (**A**) The CNV variation frequency of 33 PRG in the ESCC cohort. The height of the column represented the alteration frequency. (**B**, **C**) The mutation frequency and classification of 33 PRG. (**D**) The location of CNV alteration of 33 PRG on 23 chromosomes in the ESCC cohort.

We delved into the mutation landscape of 33 Pyroptosis-Related Genes (PRGs) annotated in the TCGA cohort. To begin, we unveiled the copy number variations (CNVs) of these 33 PRGs in all ESCC patients. Notably, all PRGs exhibited either amplification or deletion in their copy numbers. Among them, GSDMA exhibited the highest frequency of amplification, while CASP3 displayed the highest frequency of deletion (Figure 1A). As shown in Figure 1B, 16 out of 91 (17.46%) samples had genetic mutations. Most of the mutations in PRGs had a low mutation frequency, of which only *GSDMA*, *NLRP1*, *NOD2*, *PLCG1*, *NLRC4*, *NLRP2*, and *NRLRP3* had mutation frequencies > 1%. Missense mutation stood out as the predominant variant classification, while single nucleotide polymorphisms (SNPs) emerged as the most prevalent variant type. Among the diverse SNV classes, the C > T substitution notably held the highest rank (Figure 1C). In addition, we also identified the location of CNV alterations of these 33 PRGs on chromosomes (Figure 1D).

### Differential expression of PRGs in ESCC

**Figure 2:**
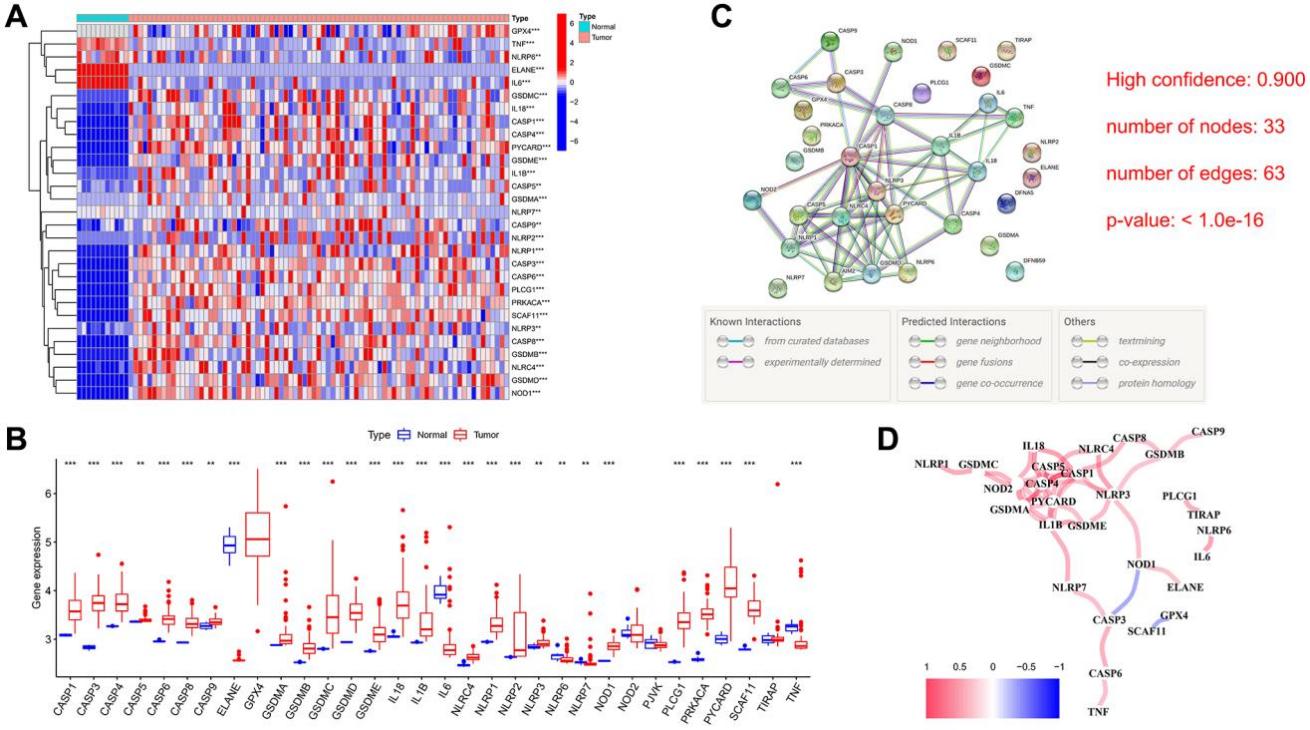
**Differential expression and interaction of 33 PRGs.** (**A**) Heatmap (blue: low expression level; red: high expression level) of PRGs between the normal and the tumor tissues. (**B**) The expression of 33 PRG in ESCC and esophageal tissues, tumor, red; normal, blue. The upper and lower ends of the boxes represented the interquartile range of values. The lines in the boxes represented median value. (**C**) PPI network showing the interactions of PRGs (interaction score = 0.9). (**D**) The correlation network of PRGs (red line: positive correlation; blue line: negative correlation. The depth of the colours reflects the strength of the relevance). ^**^P < 0.01, ^***^P < 0.001.

Upon comparing the gene expression levels of 33 potential regulatory genes (PRGs) within the TCGA cohort, we successfully discerned the presence of 29 genes exhibiting differential expression (DEGs) with a statistical significance of *P* < 0.01 (Figure 2A, 2B). We explored the expression of the 33 PRGs in ESCC and normal esophageal tissues using the TCGA-ESCC dataset. Among them, a total of 24 genes, namely CASP1, CASP3, CASP4, CASP5, CASP6, CASP8, CASP9, GSDMA, GSDMB, GSDMC, GSDMD, GSDME, IL18, IL1B, NLRC4, NLRP1, NLRP2, NLRP3, NLRP7, NOD1, PLCG1, PRKACA, PYCARD, and SCAF11, exhibited significant enrichment in the tumor group. In contrast, 5 other genes, namely ELANE, IL6, NLRP6, TNF, and GPX4, demonstrated down-regulation. In order to delve deeper into the interactions of these PRGs, we performed a Protein-Protein Interaction (PPI) network analysis, utilizing a minimum interaction score threshold of 0.9 (Figure 2C). In addition, we demonstrated a correlation network that included all the PRGs. Interestingly, only *NOD1*, *CASP3*, *GPX4*, and *SCAF11* were negatively correlated (Figure 2D).

### Functional enrichment analysis of PRGs

**Figure 3:**
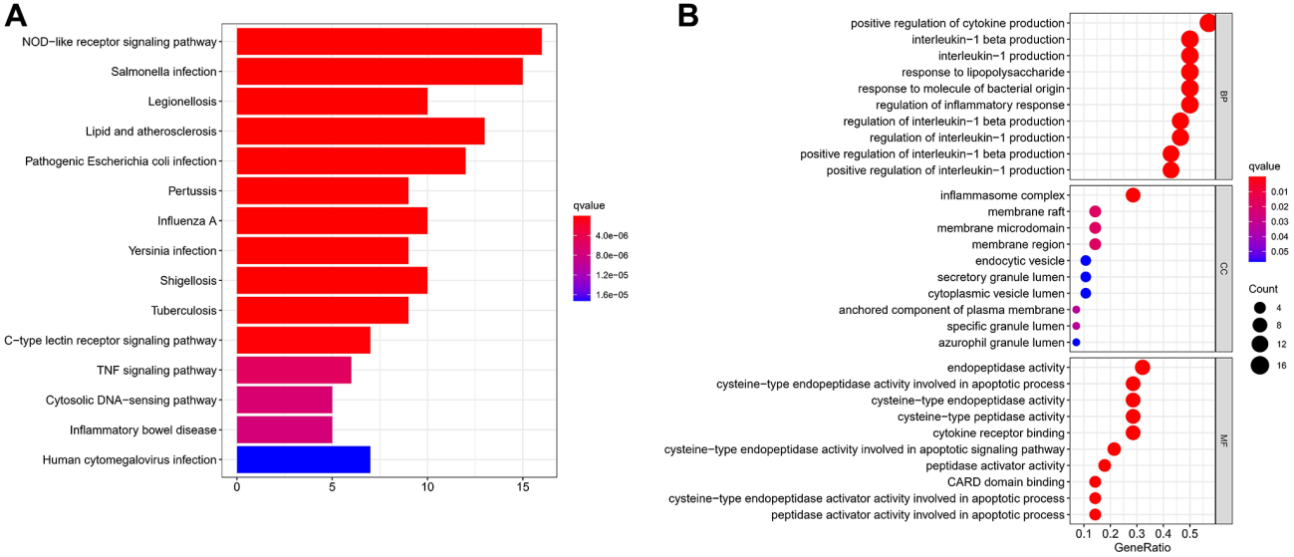
**The functional enrichment analysis of PRG in ESCC.** (**A**) Bar plot of KEGG analyses (the longer bar means the more genes enriched, and the increasing depth of red means the differences were more obvious). (**B**) Bubble plots of GO analyses (the bigger bubble means the more genes enriched, and the increasing depth of red means the differences were more obvious; *q*-value: the adjusted *p*-value).

To elucidate the biological functions of the PRGs beyond their involvement in pyroptosis regulation, we conducted Gene Ontology (GO) and Kyoto Encyclopedia of Genes and Genomes (KEGG) enrichment analyses. The KEGG analysis revealed that the 33 PRGs exhibited significant enrichment in various pathways, prominently including the NOD-like receptor signalling pathway and salmonella infection pathway, among others (Figure 3A). The gene ontology (GO) analysis revealed a predominant emphasis of the 33 Potentially Related Genes (PRGs) on cytokine production, particularly interleukin-1 production (Figure 3B).

### Construction and validation of risk model

**Figure 4:**
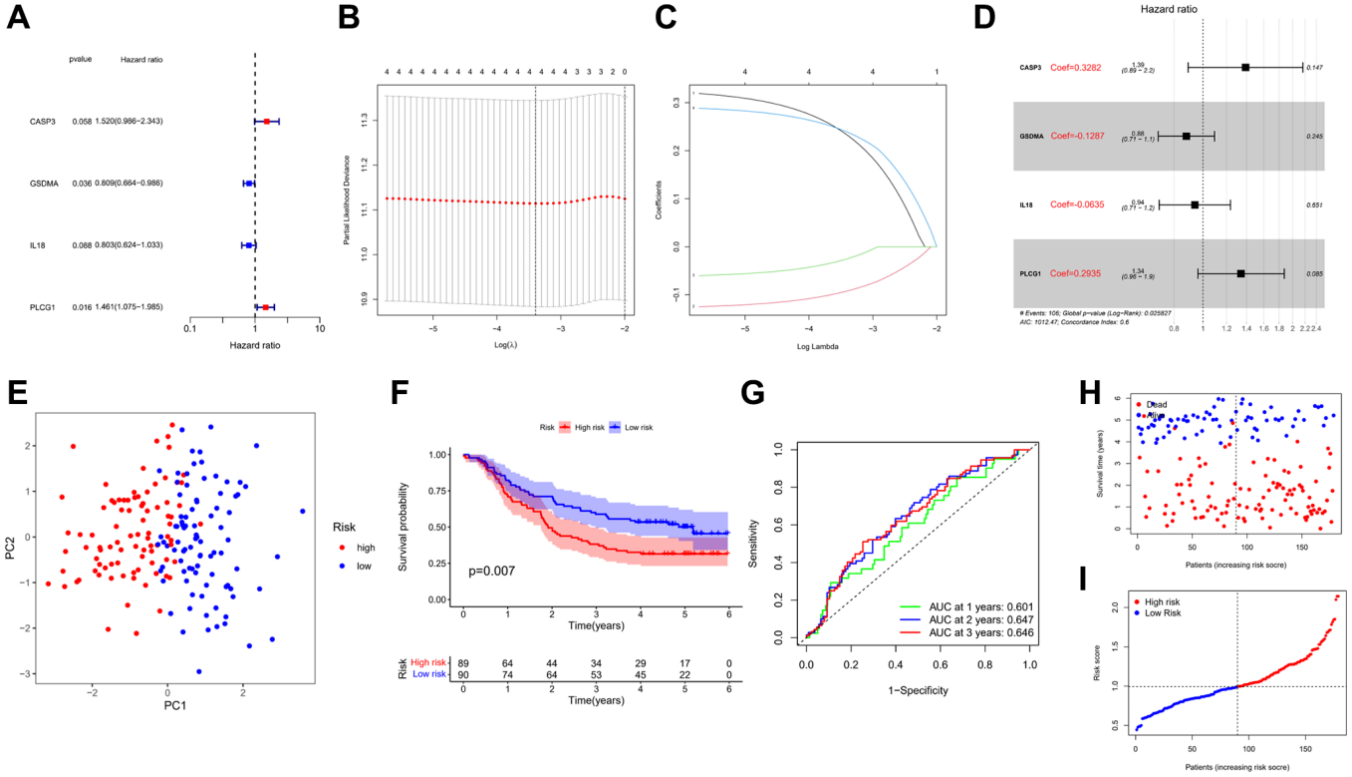
**Development of prognostic model.** (**A**) Forest plot of univariate Cox regression. (**B**, **C**) LASSO regression algorithm and cross-validation. (**D**) Forest plot of multivariate Cox regression. (**E**) PCA plot based on the risk score. (**F**) Kaplan-Meier curves for the OS of patients in the high- and low-risk groups. (**G**) ROC curves demonstrated the predictive efficiency of the risk score. (**H**) The survival status for each patient. (**I**) Distribution of patients based on the risk score.

The transcriptome data and survival information were matched, and the GEO cohort was finally included in 179 ESCC patients for modeling. Univariate Cox regression analysis (*P* < 0.1) was employed to initially screen survival-related genes, and 4 genes (*IL18*, *CASP3*, *GSDMA*, and *PLCG1*) were identified (Figure 4A). Utilizing LASSO and multivariate Cox regression analyses, we diligently crafted a signature-based model, comprising four prognostic-related genes (PRGs), selected based on the optimal λ value (Figure 4B–4D). The calculation of the risk score was executed through the following methodology: risk score = (0.3282 × *CASP3* expression) + (0.1287 × *GSDMA* expression) + (0.0635 × *IL18* expression) + (0.2935 × *PLCG1* expression). Moreover, survival risk plots and scatter plots (Figure 4H, 4I) also showed that 179 patients fell into low- and high-risk subgroups. Based on principal component analysis (PCA), patients with different risks were classified into two clusters (Figure 4E). There was a significant difference in OS time between the low- and high-risk groups (*P* = 0.007, Figure 4F). Patients assigned to the high-risk group exhibited a higher frequency of mortality and experienced shorter overall survival durations in comparison to individuals allocated to the low-risk group. The performance characteristics of the prognostic model were subsequently assessed by means of time-dependent receiver operating characteristic (ROC) analysis, yielding area under the curves (AUCs) of 0.601, 0.647, and 0.646 at 1, 2, and 3 years, respectively. These values serve as indicators of the sensitivity and specificity of the model (Figure 4G). Finally, we performed Kaplan-Meier analysis on the above 4 PRGs involved in modelling in GEO and TCGA cohorts, respectively (Supplementary Figure 1).

**Figure 5:**
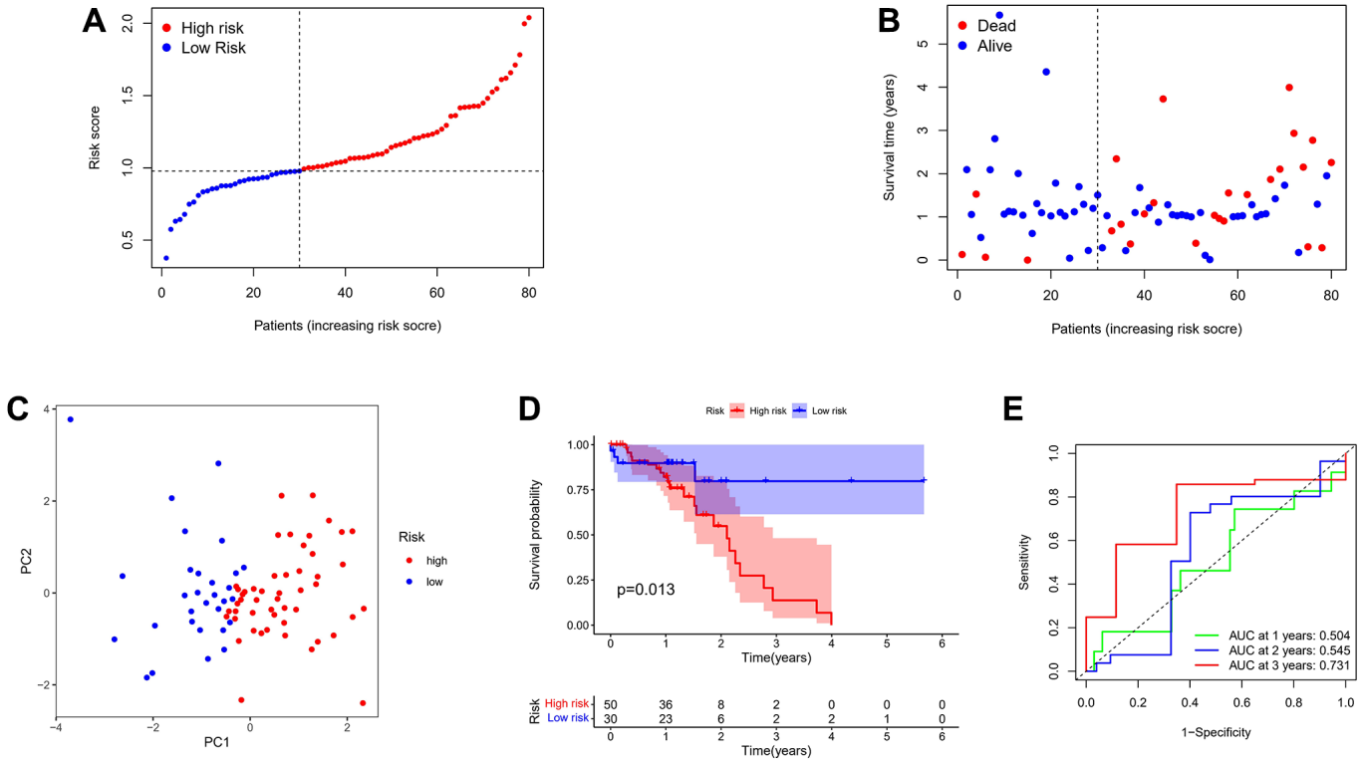
**Validation of the risk model in the TCGA cohort.** (**A**) Distribution of patients in the TCGA cohort based on the median risk score in the GEO cohort. (**B**) The survival status for each patient. (**C**) PCA plot. (**D**) Kaplan-Meier curves for the OS of patients in the high- and low-risk groups. (**E**) Time-dependent ROC curves.

A subset of 80 esophageal squamous cell carcinoma (ESCC) patients from the TCGA cohort was employed. Prior to conducting further analysis, the gene expression data underwent standardization using the “sva” package, ensuring consistency and comparability in subsequent analyses. (Supplementary Figure 2). Based on the median risk score of GEO cohort, 30 patients in TCGA cohort fell into the low-risk group, and 50 patients were classified into the high-risk group (Figure 5A). Within the low-risk subgroup of patients (as depicted in Figure 5B, on the left side of the dotted line), notably extended survival times and reduced mortality rates were observed in contrast to the high-risk subgroup. The principal component analysis (PCA) demonstrated a satisfactory degree of separation between the two aforementioned subgroups (Figure 5C). Kaplan-Meier analysis also revealed a significant difference in the survival rate between the low- and high-risk groups (*P* = 0.013, Figure 5D). ROC curve analysis of the TCGA cohort showed that our model had good predictive effect (1-year AUC = 0.504, 2-year AUC = 0.545, 3-year AUC = 0.731) (Figure 5E).

### Independent prognostic value of the risk model and construction of the nomogram

**Figure 6:**
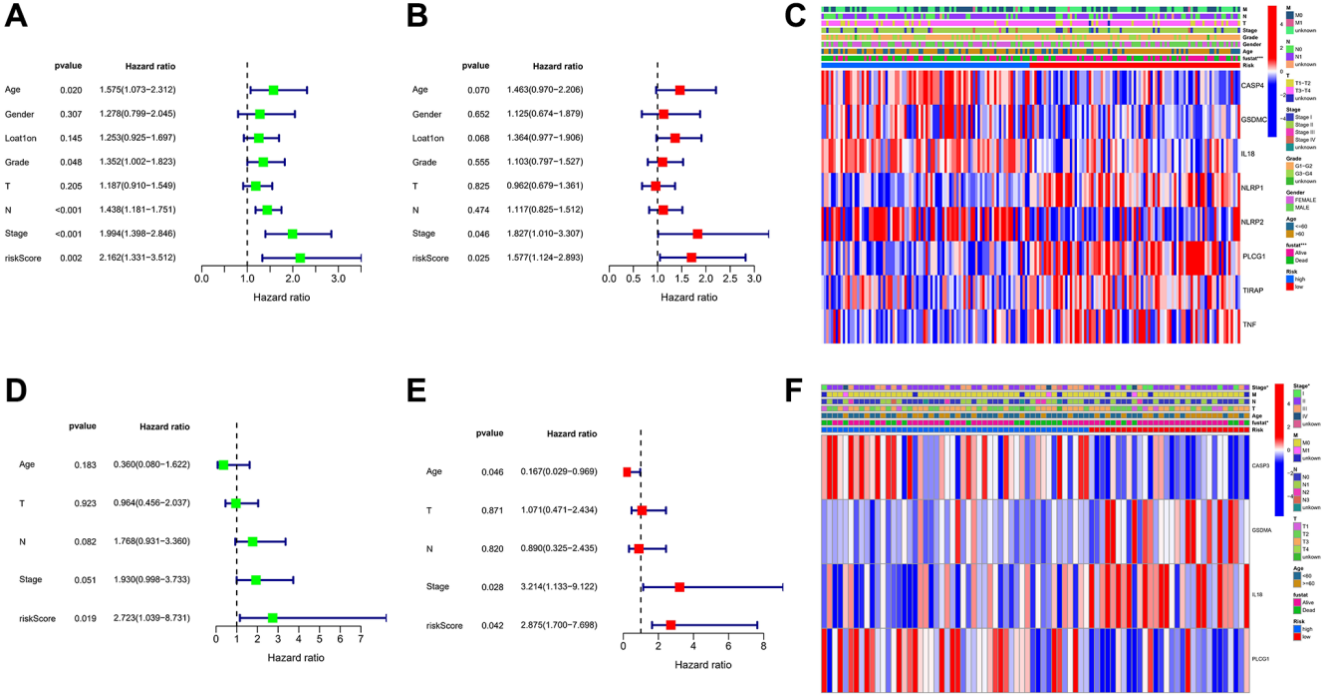
**Univariate and multivariate Cox regression analyses.** (**A**, **B**) Univariate and multivariate analysis for the GEO cohort. (**C**) Heatmap for the connections between clinicopathologic features and the risk groups in the GEO cohort. (**D**, **E**) Univariate and multivariate analysis for the TCGA cohort. (**F**) Heatmap for the connections between clinicopathologic features and the risk groups in the TCGA cohort. ^*^P < 0.05.

**Figure 7:**
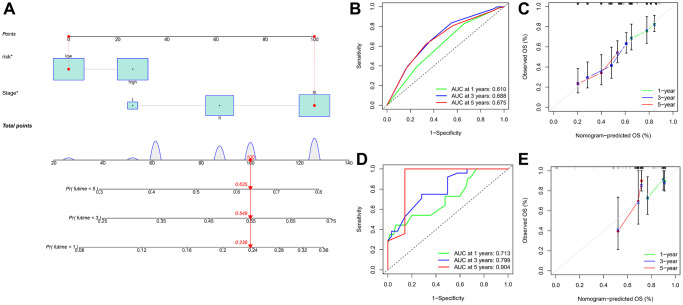
**Establishment and evaluation of a predictive nomogram.** (**A**) Nomogram based on the risk score of PRGs and clinicopathological parameters. (**B**) ROC curves of the nomogram for OS prediction at 1, 3, and 5 years in the GEO cohort. (**C**) Calibration curves of nomogram for OS prediction at 1, 3, and 5 years in the GEO cohort. (**D**) ROC curves of the nomogram for OS prediction at 1, 3, and 5 years in the TCGA cohort. (**E**) Calibration curves of nomogram for OS prediction at 1, 3, and 5 years in the TCGA cohort.

Univariate and multivariable Cox regression analyses were employed to assess whether the risk score could function as an independent prognostic factor. The results showed that the risk score was an independent prognostic factor in all cohorts (Figure 6A, 6B, 6D, 6E). Furthermore, we generated heatmaps illustrating the clinical characteristics for both the GEO and TCGA cohorts. Notably, the survival status of patients within the GEO cohort exhibited considerable heterogeneity when stratified into low-risk and high-risk subgroups. Additionally, in the TCGA cohort, there were significant disparities in the survival status and tumor stage between the low-risk and high-risk subgroups. These observations further support the discriminative power and potential clinical relevance of the risk score in predicting patient outcomes (Figure 6C, 6F). Additionally, the amalgamation of multivariate regression analysis findings within the GEO cohort culminated in the development of a nomogram designed to prognosticate survival probability (Figure 7A). The calibration curve and ROC analysis revealed that compared with the ideal model, the 1-year, 3-year, and 5-year OS rates can be predicted relatively well in the two cohorts (Figure 7B–7E).

### Comprehensive analysis of immunity and potential drugs

**Figure 8:**
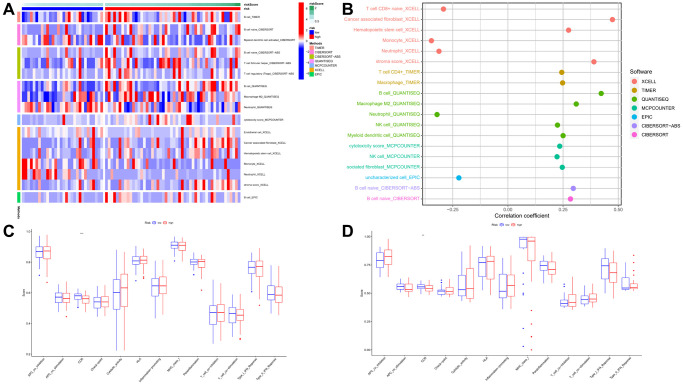
**Immunoinfiltration analysis.** (**A**, **B**) Heatmap for immune responses based on XCELL, TIMER, QUANTISEQ, MCP-counter, EPIC, CIBERSORT, and CIBERSORTABS algorithms among high- and low-risk groups. (**C**, **D**) Comparison of the enrichment scores of 13 immune-related pathways between low- and high-risk group in the TCGA cohort and GEO cohort. *P* values were showed as. Abbreviation: ns: not significant. ^**^P < 0.01; ^***^P < 0.001.

**Figure 9:**
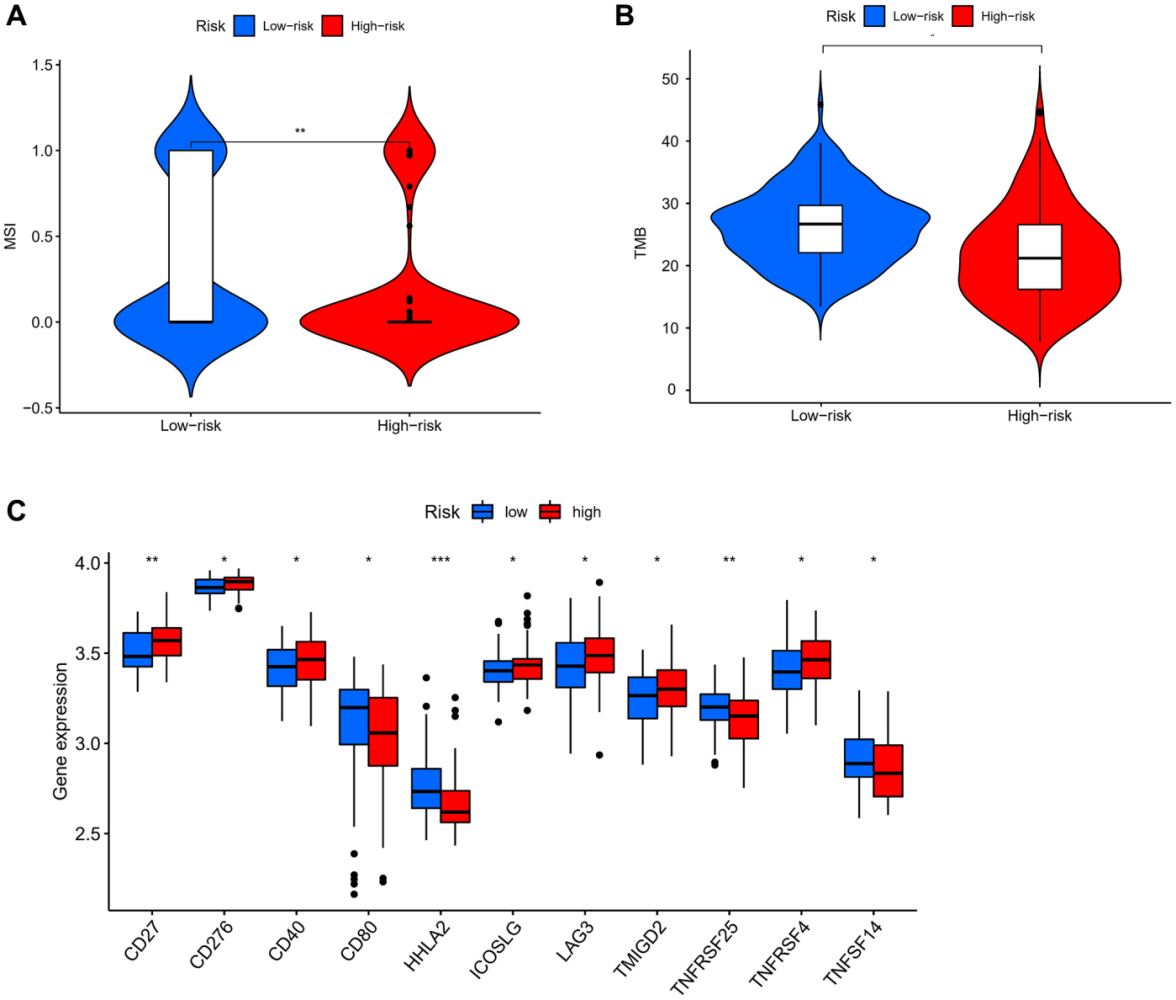
**Analysis of TMB, MSI, and immune checkpoints.** (**A**) MSI analysis in different groups. (**B**) TMB analysis in different groups. (**C**) Expression of immune checkpoints among high- and low-risk groups. *P* values were showed as. Abbreviation: ns: not significant. ^*^P < 0.05; ^**^P < 0.01; ^***^P < 0.001.

The heatmap of immune infiltration based on the XCELL, TIMER, QUANTISEQ, MCP-counter, EPIC, CIBERSORT, and CIBERSORTABS algorithms is shown in Figure 8A and Supplementary Table 1. Furthermore, we sought to investigate the association between the risk score and immune cell infiltration, particularly focusing on the correlation coefficients, was shown in Figure 8B and Supplementary Table 2. Above results showed that the proportions of tumor-infiltrating immune cells between the high-risk and low-risk groups were significantly different (*P* < 0.05). Moreover, we performed a comprehensive comparison of the activity levels pertaining to 13 immune-related pathways between the low-risk and high-risk groups within both the TCGA and GEO cohorts, employing the single-sample Gene Set Enrichment Analysis (ssGSEA) approach. Interestingly, the CCR pathway was significantly different between two cohorts. This may indicate that in patients with ESCC, there was an inseparable relationship between pyroptosis and the immune CCR pathway (Figure 8C, 8D). Furthermore, acknowledging the significance of microsatellite instability (MSI) and tumor mutational burden (TMB) in the context of tumor immunotherapy, a comparative analysis was conducted to assess the distinctions in TMB and MSI between the different risk groups. The findings demonstrated that the low-risk group exhibited notably higher MSI and TMB scores. (Figure 9A, 9B). To further explore the association between the immune checkpoints and the two risk groups, a differentiation analysis was conducted for the expression of 46 common immune checkpoints. As shown in Figure 9C, in the high-risk group, we observed a significant up-regulation of CD27, CD276, CD40, ICOSLG, LAG3, TMIGD2, and TNFRSF4. Conversely, in the low-risk group, we noted a significant up-regulation of CD80, HHLA2, TNFRSF25, and TNFSF14. These differential gene expressions signify distinct immunological profiles between the two risk groups. Moreover, exploring the association between gene expression and existing drugs is necessary to develop new therapeutic targets. Within this study, the drug-sensitivity analysis uncovered a negative correlation between the expression levels of the prognostic-related genes (PRGs) utilized in the model (IL18, CASP3, GSDMA, and PLCG1) and the majority of drugs present in the cancer therapeutic response portal database. This finding suggests that higher expression of these PRGs may potentially impede the efficacy of various drugs commonly used in cancer treatment (Supplementary Figure 3 and Supplementary Table 3).

### Validation of risk signature

**Figure 10:**
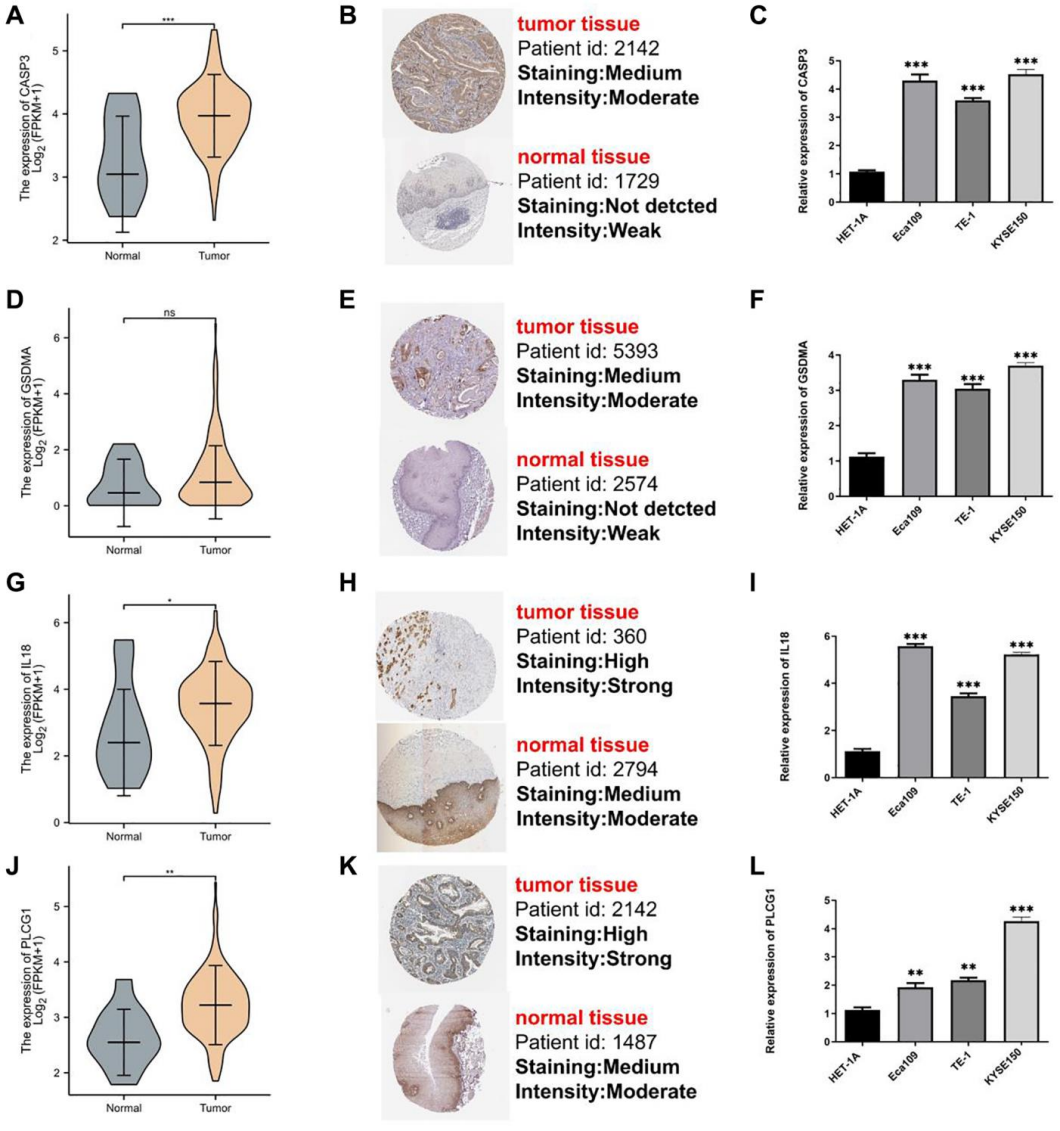
**Assays validation for genes participating in model.** mRNA expression, IHC, and qRT-PCR for CASP3 (**A**–**C**), GSDMA (**D**–**F**), IL18 (**G**–**I**), and PLCG1 (**J**–**L**). ^*^P < 0.05; ^**^P < 0.01; ^***^P < 0.001.

To further verify the expression of the four genes, we performed experimental validation. First, except for *GSDMA* (Figure 10D), the other three genes were overexpressed in the tumor samples (Figure 10A, 10G, 10J). Secondly, in the immunohistochemistry (IHC) sections available in the Human Protein Atlas (HPA) database, the protein expression levels of these genes were observed to be significantly elevated in the majority of tumor samples compared to normal esophageal epithelium. Moreover, the staining intensity was notably enhanced (Figure 10B, 10E, 10H, 10K). Finally, we validated three esophageal cancer cell lines (Eca109, TE-1, and KYSE150) and a normal esophageal epithelial cell line (HET-1A) as a control and found that all four genes were overexpressed (Figure 10C, 10F, 10I, 10L). Furthermore, the aforementioned gene expression findings in clinical tissue samples demonstrated a high degree of concordance with the RNA sequencing data analyzed through publicly accessible databases (Supplementary Figure 4A). The survival analysis conducted in our hospital cohort revealed a statistically significant association, indicating that the high-risk cohort exhibited a significantly shorter overall survival time (*P* < 0.05) (Supplementary Figure 4B).

## DISCUSSION

This study illustrated the expression and prognostic value of PRGs in ESCC. It was observed that the expressions of *CASP9*, *CASP6*, *CASP8*, *CASP5*, *CASP3*, *CASP4*, *CASP1*, *GSDME*, *GSDMB*, *GSDMA*, *GSDMD*, *GSDMC*, *IL18*, *IL1B*, *NLRC4*, *NLRP7*, *NLRP3*, *NLRP2*, *NLRP1*, *NOD1*, *SCAF11*, *PRKACA*, *PYCARD*, and *PLCG1* were increased, whereas the expressions of *ELANE*, *IL6*, *NLRP6*, *TNF*, and *GPX4* were decreased in ESCC compared to that in normal tissues. Prognostic analysis revealed that the poor survival rate of patients with ESCC is closely associated with the high expression of *CASP3*, *GSDMA*, *IL18*, and *PLCG1*. Ye et al. [15] revealed that a high expression of *CASP3*, *GSDMA*, and *PLCG1* was related to poor prognosis and immune infiltration in ESCC. Nevertheless, the two clusters generated by the consensus clustering analysis based on the DEGs exhibited no significant differences in OS. Functional enrichment analysis of PRGs was also conducted, revealing that these 33 PRGs primarily took part in cytokine production, interleukin-1 production, and the NOD-like receptor signalling pathway. Utilizing LASSO regression and Cox regression analysis, we successfully constructed a 4-gene model capable of classifying all patients diagnosed with esophageal squamous cell carcinoma (ESCC) in the GEO cohort into distinct low- and high-risk groups. The low-risk group of patients with ESCC demonstrated markedly higher survival rates compared to the high-risk group. To validate the robustness of the prognostic signature established, we further examined its efficacy in the TCGA cohort. According to the Cox regression analysis results, the PRG signature was an independent prognostic factor for ESCC. According to a predictive nomogram, the one-, three-, and five-year OS rates could be forecasted relatively well by comparing with an ideal model in the whole cohort.

Our study generated a signature featuring 4 PRGs (*CASP3, GSDMA, IL18*, and *PLCG1*) and observed that it could predict OS in patients with ESCC. *CASP3* is considered to be a mediator of pyroptosis, which causes cell pyroptosis by lysing and activating gasdermin. Luo et al. [21] suggested that the knockdown of *HOXC13* halted proliferation and caused apoptosis of ESCC cells by upregulating *CASP3*. The N-terminal domain of human *GSDMA* can form pores in the cell membrane and induce pyroptosis-like features [22]. Saeki et al. [23] revealed that *GSDMA* is a tumor suppressor gene in related studies on tumor cells. *GSDMA* is not only inhibited in esophageal cancer and gastric cancer cells, but it has been found that this mechanism can also induce cell death by upregulating the expression of GSDMA through the transcription factor LMO1.

Previous investigations have revealed a strong correlation between the proinflammatory effects of pyroptosis and the modulation of the tumor immune microenvironment (TIME) [24]. Within the tumor immune microenvironment (TIME), neoplastic cells are enveloped in an intricate interplay of chemokines, cytokines, stromal cells, and metabolites, which endow them with the capability to elude eradication [25]. Through the liberation of damage-associated molecular patterns (DAMPs) subsequent to cellular osmotic lysis, pyroptosis possesses the capacity to remodel the tumor immune microenvironment (TIME) into an immunostimulatory state, thus obstructing the proliferation and metastasis of neoplastic cells. However, in the presence of inflammatory factors, it can also facilitate the expansion of tumor cells. [26]. Recent research has demonstrated that tumor-infiltrating lymphocytes (TILs) upregulate the secretion of IL-18 through inflammasomes [27, 28]. This upregulation elicits a safeguarding pro-inflammatory effect by stimulating the production of myeloid-derived suppressor cells (MDSCs), consequently expediting the progression of the tumor [29]. PLCG1 has conventionally been acknowledged as an executor of apoptosis, operating via phosphokinase-mediated signal transduction. Nevertheless, this molecule also assumes a pivotal regulatory function in macrophage differentiation and the inflammatory response, consequently exerting control over the tumor microenvironment [30].

In our prognostic model, we quantified the functionality of immune cells and signalling pathways within tumor samples. Through this study, we unveiled a positive correlation between the risk score determined by our model and the infiltration of distinct immune cell subtypes. These findings validate earlier studies indicating that patients with high-risk ESCC, characterized by elevated levels of immune cell infiltration, exhibit a poorer prognosis [31]. The obtained results suggest that pyroptosis plays a partial regulatory role in the TME. Furthermore, our investigation revealed a noteworthy enrichment of the CCR pathway in the low-risk group. This observation implies an interconnected relationship between pyroptosis and the immune CCR pathway in patients with ESCC. These findings serve as a valuable reference for future studies delving into the underlying mechanisms of PRGs in ESCC.

MSI and TMB have emerged as crucial predictive biomarkers for cancer immunotherapy [32]. Given the significance of MSI and TMB in tumor immunotherapy, we conducted an analysis to compare the disparities between TMB and MSI among different groups. The findings demonstrated that the low-risk group exhibited higher MSI and TMB scores. Presently, multiple clinical trials investigating the efficacy of immune checkpoint inhibitors (ICIs) in ESCC patients are underway [33]. Through the evaluation of the correlation between the risk score and the expression levels of crucial immune checkpoints, it was noted that a majority of immune checkpoints (7 out of 11) displayed heightened expression in the high-risk group. This suggests that patients with higher risk scores may potentially derive greater benefits from ICIs compared to those with lower risk scores. We also found that these four model-related genes were related to targeted therapies. Among these immune checkpoints, IL18 demonstrated high sensitivity to targeted therapeutics such as vemurafenib and dabrafenib. Additionally, it exhibited heightened sensitivity to chemotherapeutic agents including vinorelbine, doxorubicin, paclitaxel, and decitabine. These findings suggest that IL18 holds potential as a predictive marker for chemosensitivity.

This study had some limitations. First, the results should be further verified using other independent datasets. Second, further experimental validation can help us understand the biological functions associated with the prognostic significance of the identified PRGs in ESCC. Thirdly, the specific reasons for the altered expression of pyroptosis-related genes are not clearly understood. In our future endeavors, we plan to delve deeper into the mutation mechanism of pyroptosis-related genes. By conducting comprehensive investigations, we aim to enhance our understanding of the underlying mechanisms that govern pyroptosis and its associated genetic alterations.

In summary, we have successfully developed and validated a prognostic model utilizing the signature of PRGs in patients diagnosed with ESCC. Our study has laid a solid groundwork for future investigations exploring the intricate interplay between PRGs and the immune response in ESCC. By establishing this association, we aim to contribute to the advancement of scientific knowledge in the field and provide a solid foundation for further research in understanding the role of PRGs in modulating the immune system in the context of ESCC.

## Supplementary Materials

Supplementary Figures

Supplementary Tables
