# Supplementary material for: A novel and robust pyroptosis-related prognostic signature predicts prognosis and response to immunotherapy in esophageal squamous cell carcinoma
Source: Aging (Albany NY). 2023 Aug 9;15(15):7811–30. doi: 10.18632/aging.204946 (PMC10457042; doi:10.18632/aging.204946)
Supplement: Supplementary Figures [file aging-15-204946-s001.pdf]

SUPPLEMENTARY FIGURES

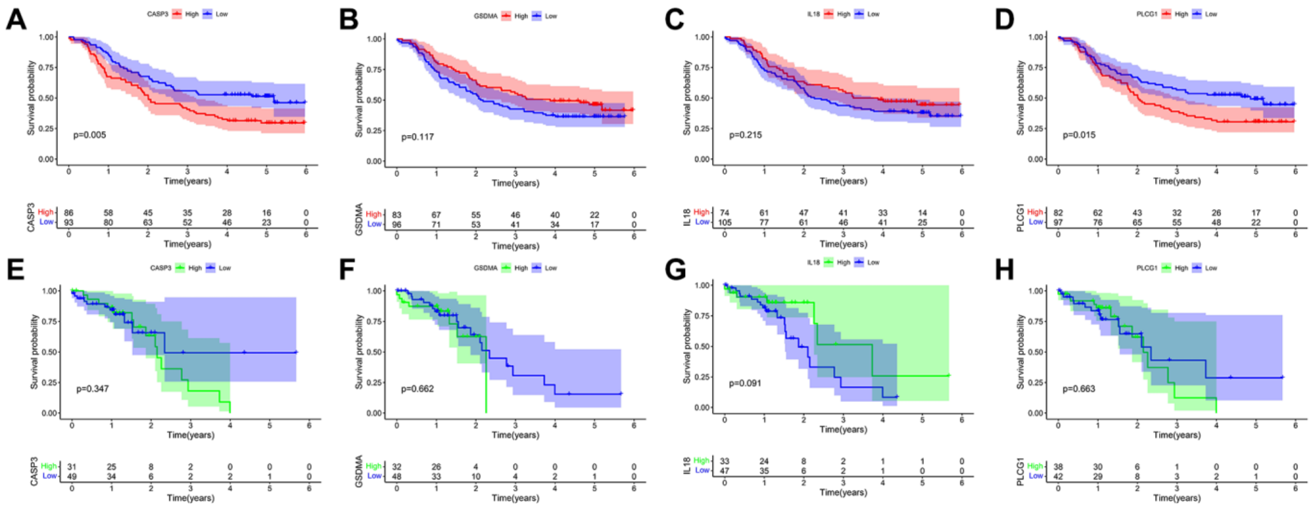

Supplementary Figure 1. The correlation between four prognostic PRG. (**A**–**D**) Survival analysis of CASP3, GSDMA, IL18, and PLCG1 in GEO cohort. (**E**–**H**) Survival analysis of CASP3, GSDMA, IL18, and PLCG1 in TCGA cohort.

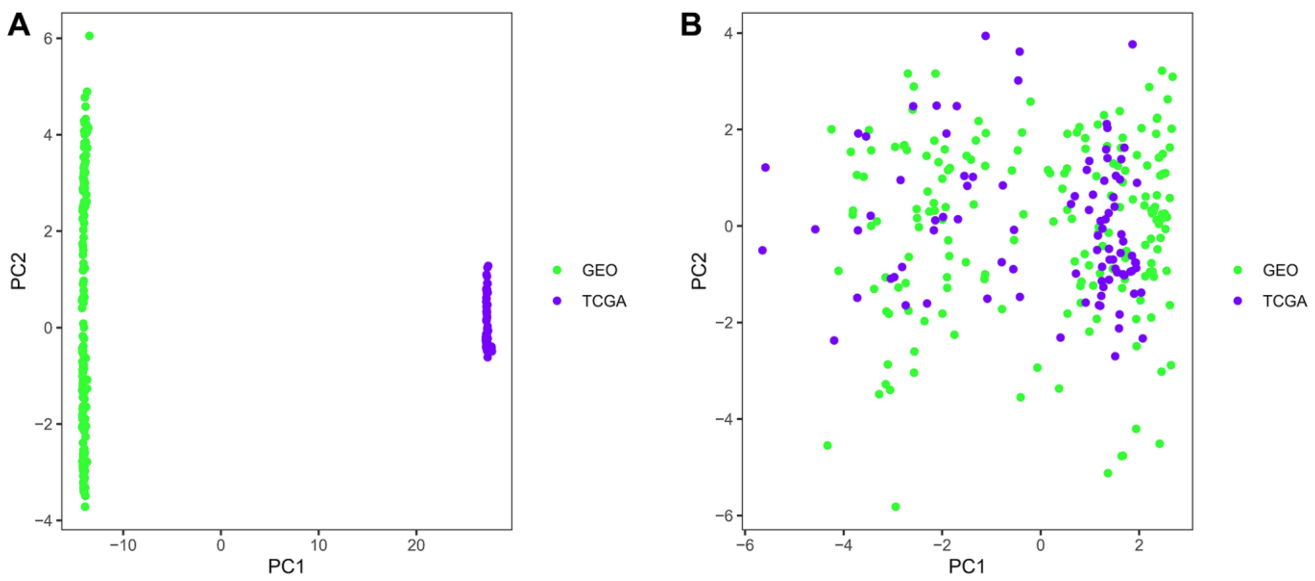

Supplementary Figure 2. Batch results of GEO and TCGA cohorts. (**A**) PCA analysis before batch. (**B**) PCA analysis after batch.

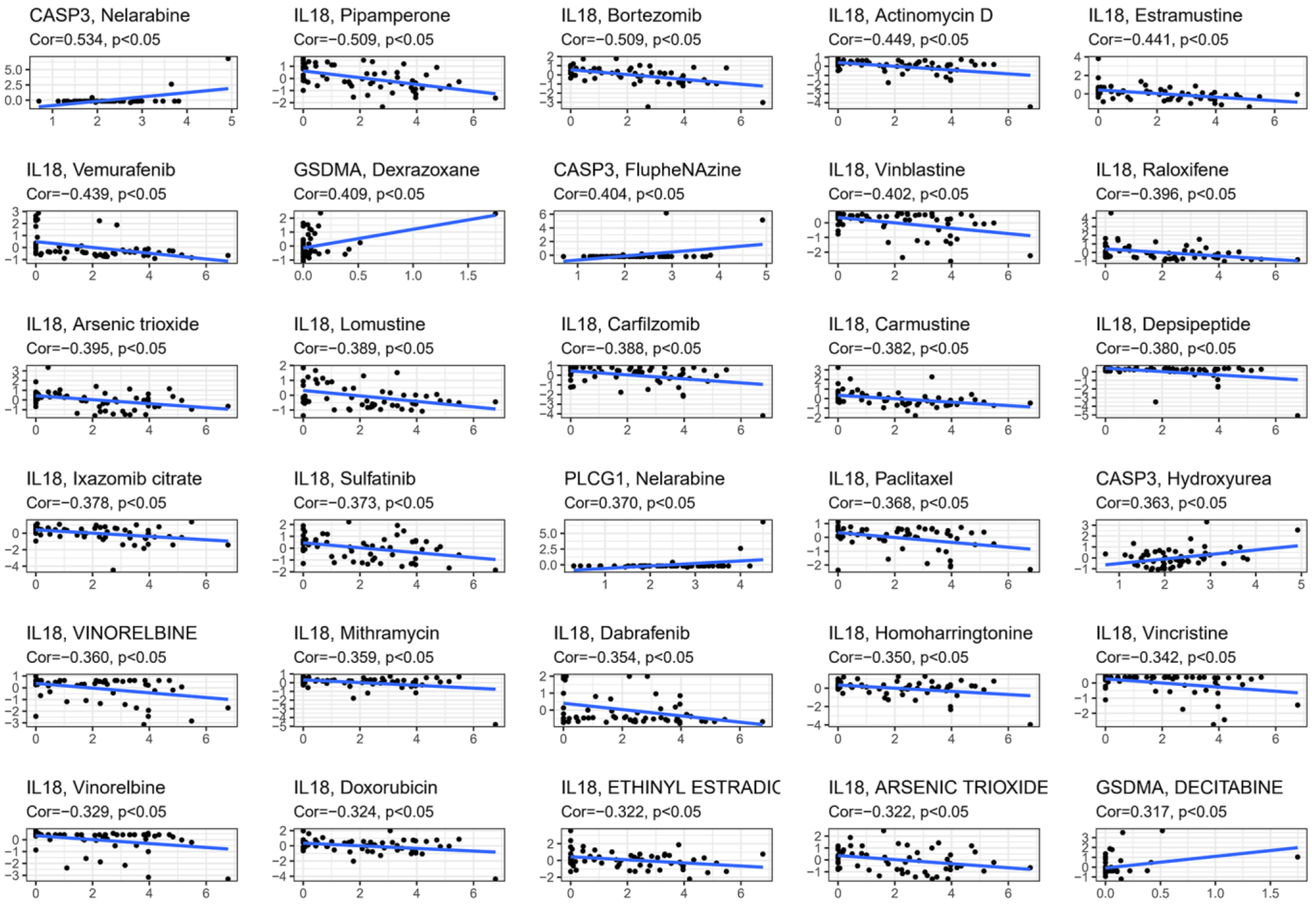

Supplementary Figure 3. Drug sensitivity.

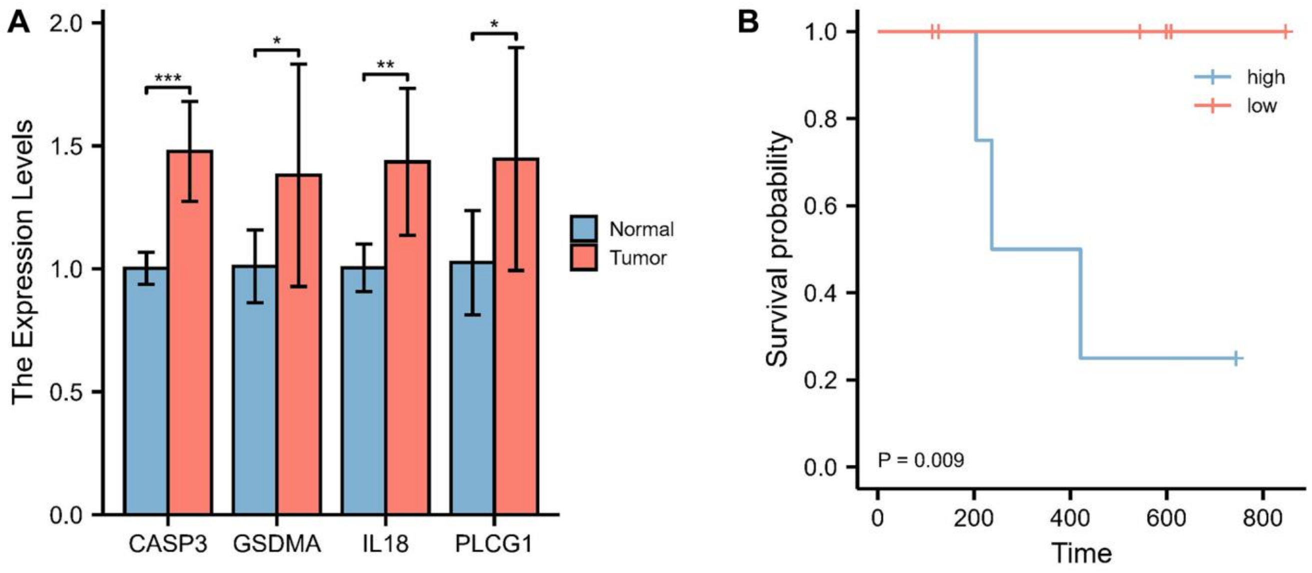

Supplementary Figure 4. Validation of risk signature in native cohort. (**A**) Survival analysis of native cohort. (**B**) qRT-PCR of the expression of 4 PRGs in 10 pairs tissues. $^{*}P < 0.05$; $^{**}P < 0.01$; $^{***}P < 0.001$.
