# Supplementary material for: A novel and robust pyroptosis-related prognostic signature predicts prognosis and response to immunotherapy in esophageal squamous cell carcinoma
Source: Aging (Albany NY). 2023 Aug 9;15(15):7811–30. doi: 10.18632/aging.204946 (PMC10457042; doi:10.18632/aging.204946)
Supplement: Supplementary Tables [file aging-15-204946-s002.pdf]

SUPPLEMENTARY TABLES

Supplementary Table 1. A list of significant immune cells based on 6 algorithms.

Immune	*P* value
B cell_TIMER	0.035561695
B cell naive_CIBERSORT	0.00650555
Myeloid dendritic cell activated_CIBERSORT	0.040464564
B cell naive_CIBERSORT-ABS	0.005395637
T cell follicular helper_CIBERSORT-ABS	0.028348751
T cell regulatory (Tregs)_CIBERSORT-ABS	0.034473007
B cell_QUANTISEQ	0.000457769
Macrophage M2_QUANTISEQ	0.008077602
Neutrophil_QUANTISEQ	0.002558986
Cytotoxicity score_MCPCOUNTER	0.032221156
Endothelial cell_XCELL	0.036042945
Cancer associated fibroblast_XCELL	0.002726991
Hematopoietic stem cell_XCELL	0.034265358
Monocyte_XCELL	0.003936186
Neutrophil_XCELL	0.005765416
Stroma score_XCELL	0.043135121
B cell_EPIC	0.023155984

Supplementary Table 2. Correlation analysis of immune cell and risk score based on 6 algorithms.

Immune	Cor	*P* value
T cell CD4+_TIMER	0.2441	0.0293
Macrophage_TIMER	0.2478	0.0267
B cell naive_CIBERSORT	0.2835	0.0108
B cell naive_CIBERSORT-ABS	0.2962	0.0076
B cell_QUANTISEQ	0.4226	0.0001
Macrophage M2_QUANTISEQ	0.3096	0.0052
Neutrophil_QUANTISEQ	−0.3211	0.0037
NK cell_QUANTISEQ	0.2247	0.0453
Myeloid dendritic cell_QUANTISEQ	0.2500	0.0253
Cytotoxicity score_MCPCOUNTER	0.2348	0.0362
NK cell_MCPCOUNTER	0.2226	0.0474
Cancer associated fibroblast_MCPCOUNTER	0.2464	0.0278
T cell CD8+ naive_XCELL	−0.2919	0.0086
Cancer associated fibroblast_XCELL	0.4736	0.0000
Hematopoietic stem cell_XCELL	0.2749	0.0136
Monocyte_XCELL	−0.3466	0.0016
Neutrophil_XCELL	−0.3123	0.0048
Stroma score_XCELL	0.3898	0.0004
Uncharacterized cell_EPIC	−0.2221	0.0479

Supplementary Table 3. Correlation analysis of expression of PRGs involved in modeling and drug.

Gene	Drug	Cor	*P* value
CASP3	Nelarabine	0.5343	0.0000
IL18	Pipamperone	−0.5090	0.0000
IL18	Bortezomib	−0.5088	0.0000
IL18	Actinomycin D	−0.4490	0.0003
IL18	Estramustine	−0.4407	0.0004
IL18	Vemurafenib	−0.4394	0.0004
GSDMA	Dexrazoxane	0.4093	0.0012
CASP3	FlupheNAzine	0.4039	0.0014
IL18	Vinblastine	−0.4024	0.0014
IL18	Raloxifene	−0.3963	0.0017
IL18	Arsenic trioxide	−0.3948	0.0018
IL18	Lomustine	−0.3894	0.0021
IL18	Carfilzomib	−0.3880	0.0022
IL18	Carmustine	−0.3823	0.0026
IL18	Depsipeptide	−0.3801	0.0027
IL18	Ixazomib citrate	−0.3776	0.0029
IL18	Sulfatinib	−0.3732	0.0033
PLCG1	Nelarabine	0.3701	0.0036
IL18	Paclitaxel	−0.3684	0.0038
CASP3	Hydroxyurea	0.3633	0.0043
IL18	VINORELBINE	−0.3605	0.0047
IL18	Mithramycin	−0.3591	0.0048
IL18	Dabrafenib	−0.3542	0.0055
IL18	Homoharringtonine	−0.3499	0.0061
IL18	Vincristine	−0.3420	0.0075
IL18	Vinorelbine	−0.3286	0.0104
IL18	Doxorubicin	−0.3237	0.0116
IL18	ETHINYL ESTRADIOL	−0.3217	0.0122
IL18	ARSENIC TRIOXIDE	−0.3216	0.0122
GSDMA	DECITABINE	0.3172	0.0135
IL18	Irofulven	0.3149	0.0142
IL18	Epirubicin	−0.3143	0.0145
IL18	Teniposide	−0.3084	0.0165
IL18	Tamoxifen	−0.3051	0.0178
IL18	Tegafur	−0.2965	0.0214
IL18	Crizotinib	−0.2962	0.0216
PLCG1	LDK-378	−0.2894	0.0249
IL18	Afatinib	0.2887	0.0253
IL18	Ixabepilone	−0.2827	0.0286
IL18	Encorafenib	−0.2827	0.0286
IL18	Dacomitinib	0.2811	0.0296
CASP3	Bendamustine	0.2802	0.0301
IL18	Abiraterone	−0.2784	0.0312
CASP3	Ifosfamide	0.2770	0.0322
PLCG1	RAPAMYCIN	0.2767	0.0323

GSDMA	Imiquimod	0.2754	0.0332
CASP3	Irofulven	−0.2748	0.0336
IL18	Erlotinib	0.2731	0.0348
IL18	Etoposide	−0.2720	0.0355
IL18	Nilotinib	−0.2709	0.0363
PLCG1	brigatinib	−0.2708	0.0363
CASP3	Lomustine	0.2706	0.0365
CASP3	Carmustine	0.2678	0.0385
IL18	Eribulin mesilate	−0.2674	0.0388
CASP3	AsparagiNAse	0.2664	0.0396
CASP3	PF-06463922	−0.2641	0.0415
CASP3	Chlorambucil	0.2636	0.0418
PLCG1	Fludarabine	0.2633	0.0421
CASP3	Calusterone	0.2595	0.0453
CASP3	Idarubicin	0.2563	0.0481
